# Molecular Analysis Uncovers the Mechanism of Fertility Restoration in Temperature-Sensitive Polima Cytoplasmic Male-Sterile *Brassica napus*

**DOI:** 10.3390/ijms222212450

**Published:** 2021-11-18

**Authors:** Qing Xiao, Huadong Wang, Hui Chen, Xiaohan Chen, Jing Wen, Cheng Dai, Chaozhi Ma, Jinxing Tu, Jinxiong Shen, Tingdong Fu, Bin Yi

**Affiliations:** 1 National Key Laboratory of Crop Genetic Improvement, Hongshan Laboratory, Huazhong Agricultural University, Wuhan 430070, China; xiaoqing04@webmail.hzau.edu.cn (Q.X.); wanghuadong@webmail.hzau.edu.cn (H.W.); 15661644378@163.com (H.C.); 17770844353@163.com (X.C.); wenjing@mail.hzau.edu.cn (J.W.); cdai@mail.hzau.edu.cn (C.D.); yuanbeauty@mail.hzau.edu.cn (C.M.); tujx@mail.hzau.edu.cn (J.T.); jxshen@mail.hzau.edu.cn (J.S.); futing@mail.hzau.edu.cn (T.F.); 2 Key Laboratory of Crop Physiology, Ecology and Genetic Breeding, Ministry of Education, Jiangxi Agricultural University, Nanchang 330045, China

**Keywords:** ambient temperature, temperature-sensitive cytoplasmic male sterility, *Brassica napus*, single-molecule long-read isoform sequencing

## Abstract

Temperature-sensitive male sterility is a heritable agronomic trait affected by genotype-environment interactions. In rapeseed (*Brassica napus*), Polima (*pol*) temperature-sensitive cytoplasmic male sterility (TCMS) is commonly used for two-line breeding, as the fertility of *pol* TCMS lines can be partially restored at certain temperatures. However, little is known about the underlying molecular mechanism that controls fertility restoration. Therefore, we aimed to investigate the fertility conversion mechanism of the *pol* TCMS line at two different ambient temperatures (16 °C and 25 °C). Our results showed that the anthers developed and produced vigorous pollen at 16 °C but not at 25 °C. In addition, we identified a novel co-transcript of *orf224-atp6* in the mitochondria that might lead to fertility conversion of the *pol* TCMS line. RNA-seq analysis showed that 1637 genes were significantly differentially expressed in the fertile flowers of 596-L when compared to the sterile flower of 1318 and 596-H. Detailed analysis revealed that differentially expressed genes were involved in temperature response, ROS accumulation, anther development, and mitochondrial function. Single-molecule long-read isoform sequencing combined with RNA sequencing revealed numerous genes produce alternative splicing transcripts at high temperatures. Here, we also found that alternative oxidase, type II NAD(P)H dehydrogenases, and transcription factor *Hsfs* might play a crucial role in male fertility under the low-temperature condition. RNA sequencing and bulked segregant analysis coupled with whole-genome sequencing identified the candidate genes involved in the post-transcriptional modification of *orf224*. Overall, our study described a putative mechanism of fertility restoration in a *pol* TCMS line controlled by ambient temperature that might help utilise TCMS in the two-line breeding of *Brassica* crops.

## 1. Introduction

Rapeseed (*Brassica napus* L.) is the third most important oil crop worldwide. Cytoplasmic male sterility (CMS), a condition of maternal inheritance in which a plant does not produce functional pollen, is widely used in three-line system crop breeding for producing F_1_ hybrids [1,2]. In rapeseed (*Brassica napus* L.), two major CMS/fertility restoration (Rf) systems have been identified and utilised: Polima (*pol*) CMS/*Rfp* and Ogura *(ogu)* CMS/*Rfo* [3]. Of these, *pol* CMS lines have been categorised into high-temperature sensitive CMS lines, low-temperature sensitive CMS lines, and temperature-stable CMS lines based on the sensitivity of male sterility to temperature [4]. At present, in China, the temperature-sensitive *pol* CMS *Brassica napus* has been successfully used in two-line system breeding and has been promoted widely [5].

Previous studies have shown that the mitochondrial gene *orf224*, co-transcribed with its downstream gene *atp6*, is related to male sterility in *pol* CMS [6]. The co-transcripts of *orf224-atp6* produce two new transcripts, 1.4 kb and 1.3 kb in size, in the presence of the *pol* CMS fertility restoration protein (RFP) [7,8,9]. An et al. used RNA-Seq to compare the gene expression profiles of fully fertile and sterile young flower buds in near-isogenic lines [10]. Researchers have discovered some candidate proteins that interact with ORF224 and RFP through the joint analysis of the transcriptome, the proteome, and the metabolome [11]. However, thermosensitive male sterility genes have not been identified, nor have the cytological characteristics or molecular mechanisms of temperature-sensitive male sterility of *pol* CMS been revealed.

Anther development involves a series of cell division and differentiation processes that are extremely sensitive to various environmental factors, including temperature [12,13,14]. In maize, hypoxia conditions act as signal molecules to trigger archesporial specification [15]. Many nuclear male sterility sites influenced by temperature or light have been identified in rice, such as *tms5* [16], *tms9-1* [17], *tms10* [18], *p/tms12-1* [19], *pms1* [20], etc. Plants usually respond to heat stress through Ca^2+^ signalling, reactive oxygen species (ROS)/nitric oxide (NO) regulation, and the unfolded protein response (UPR) pathway [21]. Excessive accumulation of ROS and misfolded or unfolded proteins are harmful to plant growth and development. In rice, heat shock proteins and the 26S proteasome remove heat-induced intracellular proteins, enhancing heat tolerance [22,23]. Misfolded proteins caused by heat stress trigger UPR in the endoplasmic reticulum (ER). UPR promotes the translocation of transcription factors located in the ER membrane to the nucleus, activating the expression of stress response genes [24,25]. In addition, heat stress can promote mRNA splicing mediated by the RNA splicing factor inositol-requiring enzyme 1 (IRE1) localised in the ER membrane [26,27]. At present, as the global climate is warming, the molecular mechanism of temperature affecting *pol* TCMS lines is critical to the application of the two-line systems in production.

Single-molecule long-read isoform sequencing (Iso-seq) based on the Pacific Biosciences (PacBio) platform generates full-length transcripts without fragmentation, and then sequencing can occur. Iso-seq technology has been previously used in *Arabidopsis* [28], rice [29], cotton [30], wheat [31], and rapeseed [32] to analyse the alternative splicing (AS) event. The RNA-seq technology can help quickly obtain the expression information of all mRNA in a specific tissue or organ in a certain state and has been widely used in the research of many animals and plants [33,34,35]. Bulked segregant analysis coupled to the whole-genome sequencing (BSA-seq) technology can help quickly locate the major genes that control extreme traits and has been widely used in gene mapping [36,37].

In this study, we aimed to investigate the phenotype and cytology of the *pol* TCMS cultivar 596 and the temperature-stable *pol* CMS line 1318. Bulked segregant analysis coupled to whole-genome sequencing (BSA-seq) was performed to identify the temperature-sensitive fertility gene loci; RNA sequencing (RNA-seq) and Iso-seq were used to compare and analyse the transcripts of 596 and 1318 at different temperatures. Then, the BSA-seq and transcriptome results were combined to identify the candidate temperature-sensitive fertility gene and to propose a mechanism of fertility restoration in *pol* TCMS.

## 2. Results

### 2.1. Flower Morphology

**Figure 1:**
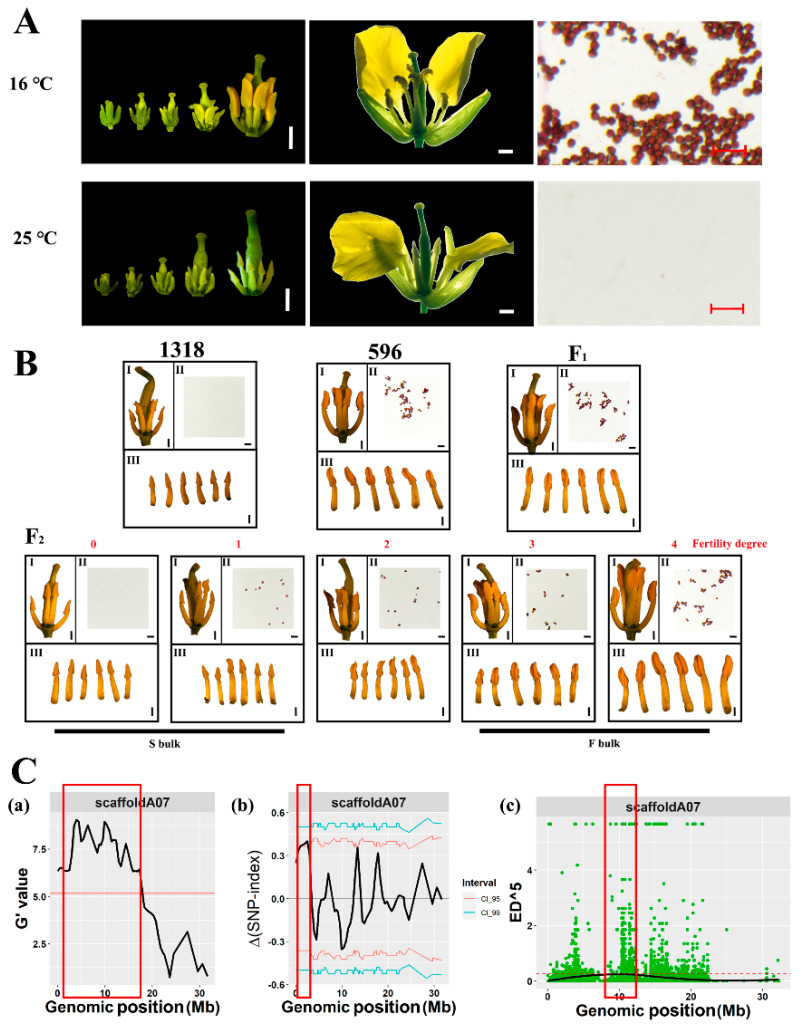
Map-based cloning of a gene controlling temperature-sensitive restoration in *pol* CMS. (**A**) Morphology of buds, flowers, and pollen grains of the *pol* TCMS plant at 16 °C and 25 °C respectively. Different stages of buds were photographed with a stereomicroscope, bars = 1 mm. Partially dissected flowers were photographed with a digital camera, bars = 1 mm. Pollen grains were stained with 1% acetocarmine staining solution, scale bars = 100 μm. (**B**) Fertility phenotype in parents and offspring. (I) Morphology of the buds, bars = 1 mm. (II) Pollen grains, bars = 100 μm. (III) Morphology of the stamen, bars = 1 mm. (**C**) BSA-seq analysis results on scaffoldA07; the red rectangle represents the predicted candidate interval. (**a**) G’ value method comparison of the differences in allele frequencies between the two extreme pools; the red line represents the threshold. (**b**) Delta SNP-index method comparison of the differences in allele frequencies between the two extreme pools. The pink line represents the 95% confidence interval, the green line represents the 99% confidence interval. (**c**) Euclidean distance method comparison of the differences in allele frequencies between the two extreme pools. The black line represents the fitted value of the differences in allele frequencies, the red dashed line represents the threshold.

**Figure 2:**
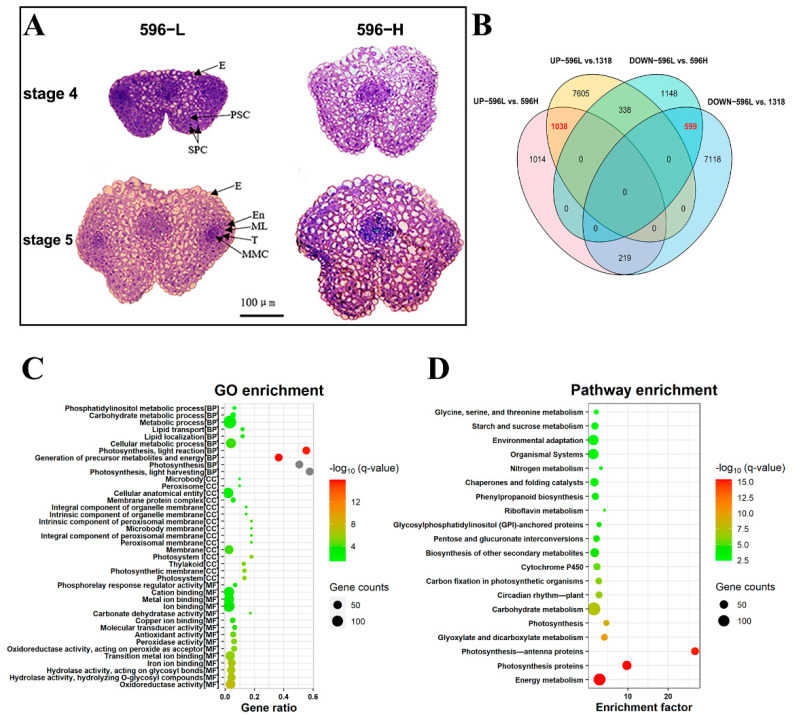
RNA-seq data analysis. (**A**) Anatomic analysis of anthers in the pol TCMS line. Bar = 100 μm. E, epidermis; PSC, primary sporogenous cell; SPC, secondary parietal cell; En, endothecium; ML, middle layer; T, tapetum; MMC, microspore mother cells [38]. (**B**) Venn plot for DEGs. (**C**) The GO enrichment for common DEGs. (**D**) The KEGG enrichment for common DEGs.

The *pol* TCMS line 596 produced fertile pollen when planted in autumn in Wuhan (30°37′ N, 114°20′ E) and sterile pollen when planted in summer in Lanzhou (36°03′ N, 103° E). To eliminate the effect of light on fertility, flowers were obtained for phenotypic and cytological observations at different temperatures under the same photoperiod in a greenhouse. At 25 °C, the *pol* TCMS line had relatively short anthers with no viable pollen grains (Figure 1A,B); the L2 layer cells did not form spore-like cells, a middle layer, an endothelium, or a tapetum at phase 4 of anther development (Figure 2A). At 16 °C, the stamen–pistil length ratio was relatively high, and the anthers developed normally with viable pollen grains (Figure 1A). Thus, male fertility was restored in the *pol* TCMS line at relatively low temperatures.

### 2.2. Identification of Temperature-Sensitive Restorer Locus

All the F_1_ plants obtained from the cross between the temperature-stable *pol* CMS line 1318 and the *pol* TCMS line 596 were fertile at an average field temperature of 16–20 °C and showed fertility restoration transition at an average field temperature of 22–25 °C, indicating that the temperature-sensitive fertility restoration genes were dominant. We investigated the fertility of the F_2_ population in the field under continuous low-temperature conditions and found that the F_2_ population showed a continuous phenotypic distribution from sterile to fertile, indicating that temperature-sensitive recovery is a quantitative trait (Figure 1B). After considering the ratio of the anther to stigma length and the number of fertile anthers, DNA from 30 extremely fertile plants (grades 3–4, F-bulk) and 30 extremely sterile individual plants (grades 0–1, S-bulk) selected from BC_1_ populations was isolated and mixed to obtain a fertile bulk and a sterile bulk, respectively. The re-sequencing of parental lines and the two bulks produced nearly 66.1 Gb of clean data (Table S1), whereas 2,819,321 high-quality single nucleotide polymorphisms (SNPs) and insertion/deletion polymorphisms (indels) were obtained by mapping reads to the ZS11v0 reference genome. Based on the G’ value, the delta SNP-index, and Euclidean distance, the temperature-sensitive restorer loci were initially located on scaffoldA07 (1.092–3.379 Mb, 7.048–10.588 Mb, Figure 1C, Supplementary Figure S1).

### 2.3. RNA-Seq and Functional Annotation of Differentially Expressed Genes (DEGs)

Flower buds of *pol* TCMS and stable *pol* CMS lines grown at 16 °C (596-L and 1318, respectively) as well as of the *pol* TCMS line grown at 25 °C (596-H) were obtained for RNA sequencing and generated 41.9 Gb of clean reads (Table S1) that aligned to the ZS11v0 reference genome. In total, 74,924 genes were expressed in the flower buds of all the groups (Table S2). A Pearson correlation coefficient heatmap and principal component analysis showed that 596-L, 596-H, and 1318 had good reproducibility among biological replicates and high discrimination between the samples (Figure S2). Of the 1038 upregulated DEGs and 599 downregulated DEGs (|log2FoldChange| ≥ 1, q ≤ 0.01) in 596-L compared with 596-H and 1318 (Figure 2B, Table S3), we randomly selected several genes for verification with a quantitative reverse transcription polymerase chain reaction (qRT-PCR) (Figure S3).

To explore the functions of DEGs, we conducted a Gene Ontology (GO) and Kyoto Encyclopaedia of Genes and Genomes (KEGG) pathway enrichment (Q-value *≤* 0.05) analysis. As a result, we found that 982 of the 1637 DEGs had GO term annotations and were significantly distributed in 10 biological processes, 18 cellular components, and 30 molecular functions (Figure 2C, Table S4). DEGs were significantly enriched in biological processes such as photosynthesis and the generation of precursor metabolites and energy. Of these, the metabolic process contained the highest number of DEGs. KEGG pathway enrichment analysis showed that 1197 DEGs participated in 191 pathways (Figure 2D, Table S4). Of these, 35 pathways were significantly enriched, including energy metabolism, photosynthesis, carbohydrate metabolism, cytochrome P450, biosynthesis of other secondary metabolites, glycosylphosphatidylinositol (GPI)-anchored proteins, riboflavin metabolism, phenylpropanoid biosynthesis, chaperones and folding catalysts, and environmental adaptation.

### 2.4. PacBio Iso-Seq

**Figure 3:**
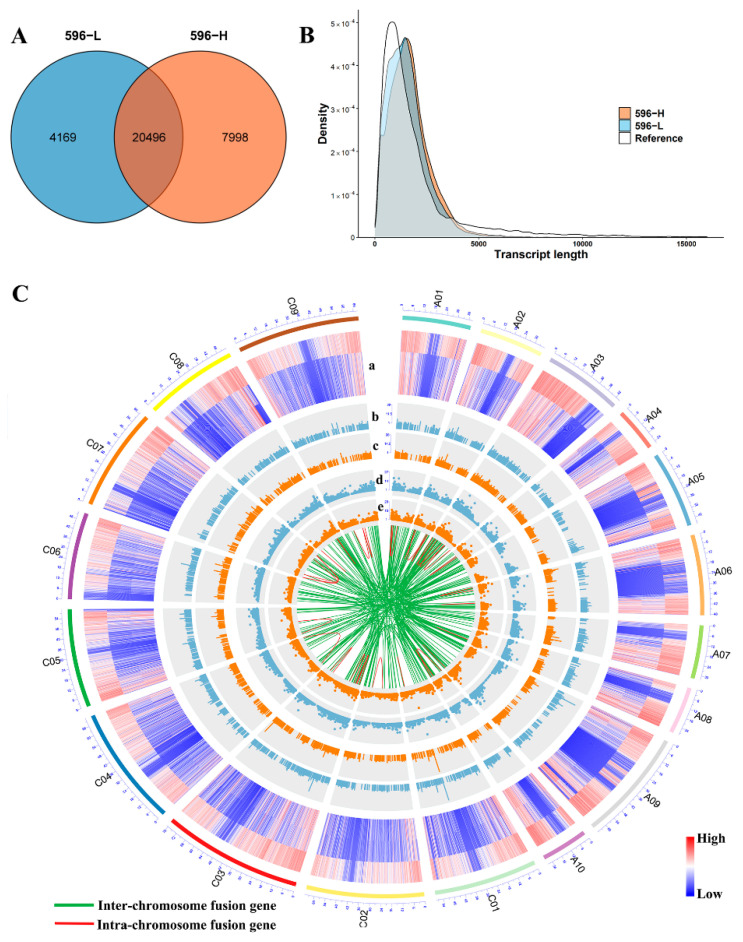
Comparison of cultivar ZS11v0 and PacBio Iso-seq data isoform annotations. (**A**) Identified isoforms in 596-L and 596-H. (**B**) Comparison of the PacBio and reference gene model length. (**C**) Circos visualisation of isoforms in the PacBio data mapping to ZS11v0. (**a**) Gene loci in different samples (top to bottom: reference, 596-L, 596-H); the gene density was counted by 100 kb (blue lines mean low gene density, red lines mean high gene density). (**b**) The histogram of the novel gene distribution in 596-L; the frequency was counted by 100 kb. (**c**) The histogram of the novel gene distribution in 596-H. (**d**) The dot plot of lncRNA distribution in 596-L, 100 kb per bin. (**e**) The dot plot of lncRNA distribution in 596-H. The lines show the fusion transcript distribution (the green lines represent the inter-chromosome fusion gene, the red lines—the intra-chromosome fusion gene).

To explore the alternative splicing (AS) of transcripts in relation to male sterility at different ambient temperatures, we prepared a cDNA library of 1–10 kb and sequenced the transcripts of 596-L and 596-H using the PacBio Iso-Seq platform. A total of 1,366,553 reads of insert (ROIs) were identified (Supplementary Table S1, Figure S4), of which 1,206,675 (88.30%) were non-chimeric full-length reads. The high-quality consensus sequences from 596-L (60,352 transcripts) and 596-H (72,368 transcripts) were mapped to the ZS11v0 reference genome, and approximately 98% of them that successfully aligned were the isoforms used in the subsequent analyses (Table S1). A total of 88,092 non-redundant isoforms mapped to the ZS11v0 reference genome covered 32,663 loci, of which 28,600 were multi-exon genes (Figure 3A). The average length of Iso-Seq transcripts was 1691 bp for 569-L and 1541 bp for 596-H, which was longer than the length of the reference annotation (Figure 3B). We identified 2867 and 2969 novel transcripts in 596-L and 596-H, respectively, via scanning using BLASTX in various protein databases. The number of annotated transcripts was 1772 in 596-L and 1861 in 596-H (Table S5). We identified 163 and 212 fusion genes in 596-L and 596-H, respectively. In total, we found 207,252 long non-coding RNAs (lncRNAs) in 596-L and 89,036 lncRNAs in 596-H. The chromosomal distribution of the genes is shown in Figure 3C. We randomly selected several novel isoforms, fusion genes, and lncRNAs for verification with RT-PCR (Figure S5). The above results enriched the annotation information of the *Brassica napus* genome.

### 2.5. AS of Transcripts at Different Ambient Temperatures

**Figure 4:**
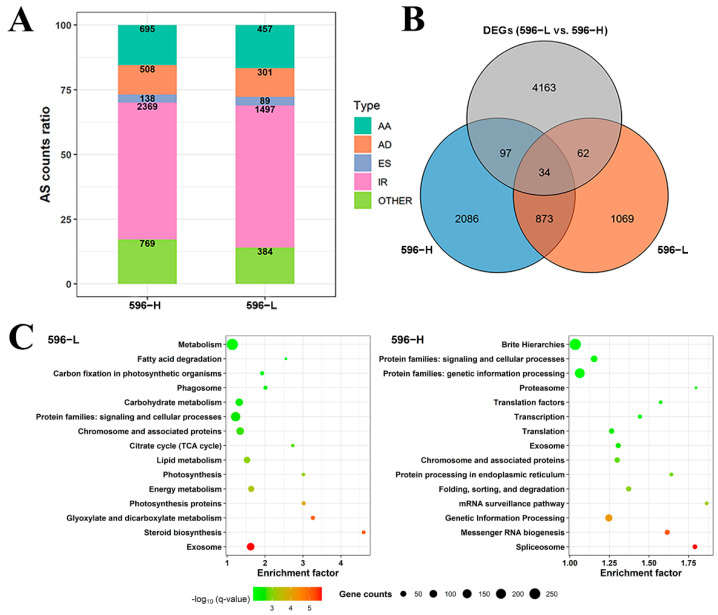
Alternative splicing analysis results. (**A**) Distribution for the alternative splicing type. (**B**) Venn plot for alternative splicing genes and DEGs. (**C**) The KEGG enrichment for specific alternative splicing genes (left: 596-L, right: 596-H).

AS of transcripts leads to structural and functional polymorphisms that increase plant diversity and adaptability. SQANTI was used to perform splice junction (SJ) statistics on non-redundant isoforms. A total of 147,594 SJs and 176,210 SJs were identified in 596-L and 596-H, respectively, of which 87.81% and 87.87% were canonical (Table S6). Using the AStalavista tools, we found 2728 AS events in 596-L, of which 54.88% showed intron retention (IR), and 4479 AS events in 596-H, of which 52.89% showed IR (Figure 4A). In addition, the AS indicator RNA cleavage factor *Ire1* was relatively highly expressed in 596-H (Figure S6). The above evidence indicates that the number and types of transcript AS increased at 25 °C. The genes involved in variable splicing under different temperatures are shown in Figure 4B, and RT-PCR was used to randomly select genes to verify the existence of variable splicing events (Figure S7). Only a few overlaps existed between variable splicing genes and DEGs (Figure 4B), indicating that the pathways in which temperature affected the transcription and post-transcriptional levels of gene regulation were different during flower bud development.

According to the GO and KEGG enrichment analyses, specific AS genes in 596-L were involved in steroid biosynthesis, glyoxylate and dicarboxylate metabolism, photosynthesis proteins, energy metabolism, photosynthesis, lipid metabolism, and citrate cycle, whereas specific AS genes in 596-H were involved in genetic information processing, messenger RNA biogenesis, mRNA surveillance pathway, protein processing in ER (protein folding, sorting, and degradation), translation, and transcription (Figure 4C and Figure S8, Table S7). Therefore, the functions of specific genes for AS in buds are different at different temperatures. These results indicate that the high temperature would increase the AS of the expressed genes in the buds, which eventually affects the development of anthers.

### 2.6. Effect of Ambient Temperature on Mitochondrial Function

**Figure 5:**
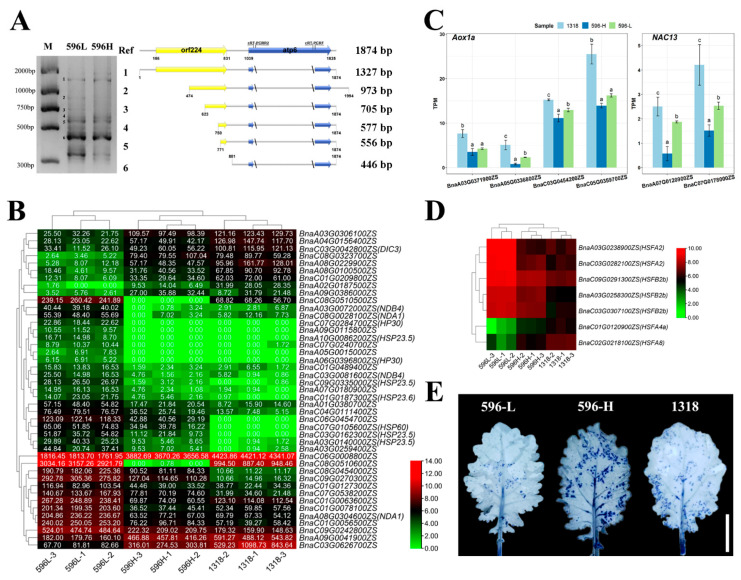
Temperature affects the mitochondrial function of the *pol* CMS line. (**A**) The transcription of *orf224-atp6* at different temperatures in the *pol* TCMS line. (**B**) Heatmap for mitochondrial localisation genes expression. (**C**) The TPM for *Aox1a* and *Nac13*. Letters indicate significant differences according to one-way ANOVA and Duncan’s test for post hoc analysis with *p* < 0.05. (**D**) Heatmap for *Hsfs* genes expression. (**E**) Superoxide anion accumulation in 596 and 1318, bar = 1 cm.

We used circularised RT-PCR (cRT-PCR) to analyse the AS of *orf244* in 596-L and 596-H since the mitochondrial co-transcript *orf224-atp6* is related to male sterility in *pol* CMS plants. The results revealed an *orf224*-*atp6* co-transcript of 1874 bp and two *atp6* transcripts of 1103 bp and 993 bp in both bulks. However, a transcript of 1520 bp with only 357 bp nucleotides of *orf224* was specifically identified in 596-L (Figure 5A), which is related to the restoration of TCMS.

The expression of nuclear-encoded mitochondrial-targeted genes was further analysed in 596-L and 596-H using RNA-seq data (Figure 5B). The results showed that *Dic3* (*BnaC03G0042800ZS*) encoding the mitochondrial dicarboxylate carrier protein was upregulated in 596-H. The gene is involved in the proton electrochemical gradient energy dissipation pathway of uncoupling proteins that protects plant cells from oxidative stress. We also found that *Nda1* (*BnaA08G0304600ZS*, *BnaC08G0028100ZS*) and *Ndb4* (*BnaA03G0072000ZS*, *BnaC03G0081600ZS*) of type II NAD(P)H dehydrogenases, as well as *Hp30* (*BnaC07G0284700ZS*, *BnaA06G0396800ZS*) that interacts with *Ndc1*, were upregulated at 596-L to regulate the redox state in the cell. In addition, the mitochondria-localised *Hsp20*-like (*BnaA10G0086200ZS*, *BnaC09G0335000ZS*, *BnaC03G0162300ZS*, *BnaA03G0140000ZS*, and *BnaC01G0187300ZS*) and *Hsp60* (*BnaC07G0105600ZS*) were upregulated in 596-L. These results indicate that mitochondrial localisation genes are involved in the response to changes in the temperature of the environment.

The young buds of 596 grown at a low temperature were treated with mitochondrial uncoupling agent carbonyl cyanide 4-(trifluoromethoxy)phenylhydrazone (FCCP). The results showed that after treating the young buds with a low concentration of FCCP, they gradually turned yellow and eventually died, indicating that the decoupling of the mitochondrial respiratory chain affects the development of buds (Figure S9). 

### 2.7. Analysis of the Genes Related to Mitochondrial Retrograde Signal Regulation

Retrograde signals are used by organelles to regulate nuclear gene expression; those from mitochondria are crucial for plant growth, development, and stress response. AOX1A (alternative oxidase 1A) is a marker of the mitochondrial retrograde reaction. Of the four copies of *Aox1a* in the ZS11v0 reference genome, *BnaA05G0336800ZS* and *BnaC05G0359700ZS* were significantly downregulated in 596-H compared with 596-L, whereas the homologous genes on A03 and C03 were not significantly different between the two bulks. Besides, *BnaA03G0371900ZS*, *BnaA05G0336800ZS*, and *BnaC03G0454200ZS* were significantly upregulated in 1318 compared with 596-L, whereas *BnaC05G0359700ZS* was not significantly different between the two bulks (Figure 5C). The expression levels of *BnaC03G0454200ZS* and *BnaC05G0359700ZS* were high in all the three bulks. We also examined the expression of *Nac13*, a positive regulatory transcription factor of *Aox1a* [39] that has two copies in the ZS11v0 reference genome. Compared with 596-L, *Nac13* was downregulated in 596-H, indicating that the lower expression levels of *Aox1a* (*BnaA05G0336800ZS* and *BnaC05G0359700ZS*) in 596-H might be influenced by *Nac13* (Figure 5C). Interestingly, *Aox1a* (*BnaA03G0371900ZS*, *BnaA05G0336800ZS*, and *BnaC03G0454200ZS*) was upregulated in 1318 compared with 596-L, whereas the expression levels of *Nac13* were not significantly different between the two bulks. Thus, the retrograde signal from the mitochondria of 1318 might activate regulatory factors other than *Nac13* that upregulate *Aox1a*.

### 2.8. Archesporial Specification Needs Appropriate ROS Levels

Transcriptome data analysis showed that some ROS-responsive heat shock transcription factors (HSF) were differentially expressed among the three bulks. Of these, redox sensors *Hsfa4a* and *Hsfa8* were upregulated in 596-H and 1318, whereas low oxygen response factors *Hsfa2* and *Hsfb2b* were upregulated in 596-L (Figure 5D). These results showed that the ROS levels might be lower in fertile flower buds in which the oxidoreductase genes were differentially expressed (Figure S10), highlighting the importance of redox balance during anther development. Staining with nitro blue tetrazolium (NBT) showed that 596-L has a low level of ROS accumulation (Figure 5E). The above results revealed that the normal development of archesporial cells could be attributed to the high redox balance ability of 596-L.

### 2.9. Identification of Candidate Genes

BSA-seq analysis revealed the presence of quantitative trait loci (QTL) on scaffoldA07 that control the temperature-sensitive fertility restoration in 596. The candidate interval contained 303 genes (Table S8). Of these, 12 genes were annotated to encode mitochondria-targeted genes; two were not expressed in flower buds (Table 1 and Table S2), whereas the remaining 10 contained 182 SNPs and indels. After screening, a total of 100 variant sites were obtained for the 10 genes on scaffoldA07 (Table S8). Of these, *BnaA07G0016300ZS*, *BnaA07G0027800ZS*, and *BnaA07G0034100ZS* that were predicted to be pentatricopeptide repeat proteins localised to the mitochondria (Figure S11) were differentially expressed in 596-L and 1318 (Figure S12).

## 3. Discussion

Temperature-sensitive male sterility is a heritable trait influenced by environmental conditions. Most types of cytoplasmic male sterility found in crops are affected by the environment, and there are few completely stable sterile lines. At present, in China, the temperature-sensitive *pol* CMS *Brassica napus* has been successfully bred and widely promoted [5]. Various genes that control the phenomenon of photo–thermosensitive nuclear male sterility have been identified and cloned in rice, and the molecular mechanism controlling the influence of light and temperature on it has thus been revealed [16,18,20]. In contrast, information on the underlying mechanism of *pol* TCMS is limited since no genes that directly control the trait have been discovered.

At present, most of the restorer genes for cytoplasmic male sterility in crops are identified as PPR genes [2,40]. PPR genes can modify the CMS transcript after its transcription [9,41,42]. In rice, the splicing of the sterile transcript *atp6-orfH79* of CMS-HL requires the participation of the PPR gene RF5 and the glycine-rich protein GRP162 [43]; another restorer gene RF6 encoding the PPR protein interacts with the glycokinase OsHXK6 to degrade the *atp6-orfH79* transcript [44]. In the study of *nap* CMS in *Brassica napus*, it was found that when the restorer gene *Rfn* is present, the production of the 1.2 kb transcript can be detected by using a *orf139* probe [45]. In *Ogu* CMS, the fertility restoration PPR-B protein specifically binds to the coding region of the sterility gene *orf138* inhibiting the translation extension of *orf138* mRNA, thereby reducing the protein content of ORF138 to achieve the purpose of fertility restoration [46]. Previous studies showed that the co-transcripts of *orf224-atp6* produce two new transcripts in the presence of pol CMS fertility restoration protein (RFP) in *pol* CMS [7,8]. This study showed that a 1.5 kb *orf224-atp6* post-transcriptional modified transcript identified in 596-L might be involved in the fertility restoration process at low temperatures (Figure 5A), which is similar to the *orf224-atp6* transcript in the restorer line in the presence of the RFP protein [9]. Therefore, we speculate that the fertility restoration process at low temperatures may be affected by other organelle-editing factors.

The organelle-editing factors discovered so far mainly include the PPR protein, MORF multicellular editing factors, etc. [47,48,49,50]. At present, there have been many examples of using BSA-seq to successfully locate and clone genes [37,51,52]. Therefore, we combined BSA-seq and RNA-seq analyses to reveal several candidate genes that might be involved in the fertility restoration process at low temperatures. Some researchers found that the P-type PPR protein CDE4 is important for splicing the chloroplast gene introns at low temperatures but has no function at high temperatures [53]. The candidate temperature-sensitive genes we identified are also PPR genes which might process the *orf224-atp6* transcript at the post-transcriptional level at a low temperature in order for *atp6* to partially perform its normal function and reduce the toxic effect of ORF224 in anthers.

The development of anthers is extremely sensitive to various environmental factors, including temperature [13]. Under high-temperature stress, flower buds produce excessive ROS, the metabolism of carbon and nitrogen in anthers changes, and the tapetum cells advance PCD, etc. [14]. Studies have shown that the excessive accumulation of ROS can lead to oxidative damage, ultimately leading to pollen abortion [54]. In maize, hypoxia conditions act as signalling molecules to trigger archesporial specification [15]. In this study, the anatomic results showed that the L2 layer cells did not undergo specification at a high temperature (Figure 2A), and staining with NBT revealed that low levels of ROS accumulated in 596-L (Figure 5E). At the same time, the RNA-seq results showed that type II NAD(P)H dehydrogenases genes *Nda1* and *Ndb4*, as well as *Hp30* that interacts with *Ndc1*, were upregulated in 596-L (Figure 5B). Type II NAD(P)H dehydrogenases can be coupled with alternative oxidase (AOX) to form a non-phosphorylated respiratory pathway, allowing the cell to escape the control of adenylate, regulating its redox balance [55]. In this study, low concentration of the mitochondrial respiratory chain uncoupling agent FCCP caused ROS to increase and the flower buds to gradually turn yellow until death (Figure S9). Therefore, the temperature-sensitive recovery of male sterility might result from regulating the redox level.

The alternative oxidase (AOX) is a mitochondrial retrograde signaling indicator that provides a degree of homeostasis signaling to the organelle by controlling the level of potential mitochondrial signaling molecules, such as superoxide and some important redox couples [56,57,58,59]. In this study, mitochondrial retrograde signal gene *Aox1a* had a high expression level at a low temperature, and the expression levels of *Hsfs*, *Hsps*, and other genes that can regulate the redox state of the cell were also high (Figure 5B–D). Therefore, these genes may be able to respond to mitochondrial signal molecules at low temperatures, thereby regulating the mitochondrial redox level and ensuring redox homeostasis during the development of anthers.

However, it is interesting that at high temperatures, the expression of these genes is affected to varying degrees. It has been reported in the literature that high temperatures can cause ER stress [24,25]. Plants can restore the protein homeostasis of the endoplasmic reticulum to a certain extent through UPR and rebuild the balance of the ER [60,61]. In *Arabidopsis*, the IRE1 protein activated by ER stress can recognise and splice mRNA, reduce protein synthesis, and relieve ER stress [62,63]. In this study, Iso-seq data analysis found that more AS types and events occur in young flower buds at high temperatures (Figure 4A). In addition, the AS indicator RNA cleavage factor *Ire1* was relatively highly expressed in 596-H (Figure S6). The endoplasmic reticulum-associated degradation (ERAD) system containing the E3 ubiquitin ligase can degrade incorrectly folded proteins and avoid the release of defective proteins [64]. Among the special AS genes of 596-H, more genes are annotated as ubiquitin ligase genes related to protein processing (Figure 4C, Table S7). Therefore, we speculate that a high temperature will induce ER stress in the young flower bud cells, which will result in the failure of the normal expression of many genes, leading to male sterility. At the same time, UPR can trigger AS of mRNA and degradation of misfolded proteins, thereby alleviating the burden and damage of a part of the endoplasmic reticulum.

Therefore, we proposed a hypothesis model for fertility transition in *pol* TCMS. At relatively low temperatures, the co-transcripts of *orf224-atp6* are cleaved by RNA-editing factors (PPR?), allowing the ATP synthase to function normally. As a result, the increased energy consumption in the process of anther development elevates ROS levels and activates mitochondrial retrograde signals, *Nac13* and some transcription factors that were transferred from the ER into the nucleus-upregulated *Aox1a*, activating mitochondrial cyanide-resistant respiration. Furthermore, *Hsfs* is activated to balance redox levels; consequently, hypoxic conditions activate the differentiation of archesporial cells (Figure S13A). At relatively high temperatures, a large number of unfolded proteins or misfolded proteins are accumulated in the cells, leading to ER stress, and RNA-editing factors are not synthesised normally, leading to a full-length translation of *orf224* and consequent mitochondrial dysfunction. Besides, transcription factors that activate *Aox1a* cannot fold properly, decreasing the energy consumption in the cyanide-resistant respiratory pathway. The cells activate the UPR pathway, increasing the AS of mRNA and accelerating the degradation of unfolded proteins. Although the uncoupling of mitochondrial unfolded proteins and ATP synthase helps balance the redox state, ROS accumulation is high in the cells, resulting in the failure of archesporial cell differentiation (Figure S13B).

## 4. Materials and Methods

### 4.1. Plant Material, Growth Conditions, and Phenotyping

The *pol* TCMS 596 and the temperature-stable *pol* CMS 1318 lines were used in this study. The 596 plants were crossed with 1318 to obtain F_1_ plants, which were self-crossed to obtain F_2_ segregation and backcrossed with 1318 to produce BC_1_ lines; 596, 1318, F_1_, and BC_1_ were planted in Wuhan, Hubei Province (from October to May), and 596, 1318, and F_1_ were planted in Lanzhou, Gansu Province (from May to August), China. Parent lines (596 and 1318) were grown in a greenhouse at 16 °C and 25 °C with a photoperiod of 16 h light/8 h dark. Pollen grains were collected before flowering and stained with a 1% acetyl carmine staining solution. Stained pollen was photographed under a microscope. Images of anthers at different stages were captured using a Nikon DS-RI1 camera (Nikon, Tokyo, Japan). Flower buds (length, 0–1 mm) were collected, frozen in liquid nitrogen, and stored at −80 °C for total RNA extraction. Three biological replicates were obtained for each sample. 

### 4.2. Anatomic Analysis

Flower buds of different lengths were vacuum-infiltrated and fixed with 2.5% (*w*/*v*) glutaraldehyde in 0.1 M phosphate buffer (pH 7.2). Fixed materials were dehydrated through a graded series of ethanol (50%, 70%, 80%, 90%, 100%, and 100%) and embedded in resin using a Technovit Embedding Kit (Germany). Semi-thin (2 μm) sections were obtained using an automatic microtome (Microm HM 360, Thermo, Waltham, MA, USA), stained with 1% toluidine blue for 10 s at 22 °C, and observed under a Nikon Eclipse 80i microscope (Nikon, Tokyo, Japan). 

### 4.3. BSA-Seq and Data Analysis

Total DNA was isolated and purified from plant leaves using a DNA Secure Plant Kit (Tiangen, Beijing, China). DNA quality and quantity were assessed using a Nanodrop 2000 (Thermo Scientific, Waltham, MA, USA) and an Invitrogen Qubit 2.0 (Thermo Fisher Scientific, Waltham, MA, USA). DNA that passed the quality threshold was constructed using the standard Illumina library preparation procedures and sequenced using an Illumina Hiseq 2000 with a paired-end read length of 2 × 150 bp (Illumina, San Diego, CA, USA). We used FastQC (http://www.bioinformatics.babraham.ac.uk/projects/fastqc/, accessed on 10 November 2021) to check the quality of the raw data read and Trimmomatic [65] to obtain clean data. The reads were aligned to the ZS11.v0 reference genome [66] using Bowtie 2 [67] with the default parameters. Picard was used to remove PCR duplicates, Bowtie2 (L, –0.3, –0.3) was used to realign the reads to the reference genome, and SAMTools [68]—to sort the paired-end readings. The parameters for the Genome Analysis Toolkit (GATK, v3.6-0-g89b7209) for SNP/indel calling were as follows: QUAL < 30.0, MQD < 13.0, FS > 20.0, MQ < 20.0, MQranksum < –3.0, and ReadPosRankSum < –3.0. The screening parameters for SNPs were as follows: (1) homozygous parental genotypes; (2) different genotypes among the parents; (3) 1318 and S-bulk were the same genotypes; and (4) minimum depth = 10, maximum depth = 100. To identify QTL, QTLseqr [69] was used to calculate the G’ value with a threshold of the false discovery rate equal to 0.01 and the delta SNP-index value with a window size of 1 Mb. To identify QTL, the Euclidean distance of the fifth power was used for Loess regression fitting, and the threshold value was set as the median value plus three times the standard deviation. The SNPs were annotated using SnpEff [70] and the Integrative Genomics Viewer (IGV) [71] was used to display information on genetic variations.

### 4.4. RNA Preparation and Sequencing

Total RNA was isolated and purified using an RNAprep Pure Plant Plus Kit (Tiangen, Beijing, China) according to the manufacturer’s instructions. RNA purity, concentration, and integrity were assessed using a Nanodrop 2000 and an Agilent 2100 Bioanalyser (Agilent Technologies, Santa Clara, CA, USA). Qualified RNA was constructed using the standard Illumina library preparation procedures and sequenced using the Illumina NovaSeq 6000 with a paired-end read length of 2 × 150 bp. Equal amounts of RNA from 596-L and 596-H were mixed for building the Iso-seq library that was prepared according to the standard protocol (https://www.pacb.com/, accessed on 10 November 2021). The mRNA was reverse-transcribed using a SMARTer PCR cDNA Synthesis Kit (Takara, Tokyo, Japan). The PCR products were used to generate the SMRTBell library, and fragments of 1–10 kb were selected to obtain the sequencing library on a PacBio Sequel II platform. 

### 4.5. Illumina Data Analysis

FastQC was used to test the quality of reads and the size of adapter sequence contamination in raw data, whereas Trimmomatic was used to obtain clean data. Clean reads were aligned to the ZS11v0 reference genome using HISAT2 [72]. Perl script was used to obtain perfect reads with paired-end and unique matches on the genome. SAMtools were used to sort the paired-end reads and estimate the gene expression level using featureCounts [73]. R was used to calculate the Pearson correlation coefficient and for principal component analysis. Transcripts per million (TPM) were used to identify the expression of each gene, and differential expression analysis was performed with R with the DESeq package [74]. Genes with fold changes > 2 and q-values < 0.01 were considered DEGs. The GO and KEGG enrichment analyses (q < 0.05) were performed using TBtools [75]. The significant differential results for gene expression among the samples was obtained according to one-way ANOVA and Duncan’s test for post hoc analysis with *p* < 0.05 using SPSS (Statistical Product and Service Solutions v26.0).

### 4.6. PacBio Data Analysis 

SMRT-Analysis 5.1.0 (http://www.pacb.com/products-and-services/analytical-software/smrt-analysis/, accessed on 10 November 2021) was used to obtain high-quality, full-length isoforms. Subreads with fragment length < 300 bp or accuracy < 0.75 were excluded from the sequencing data. Reads with full passes > 0 and minimum predicted accuracy = 75 were considered ROIs (reads of insert). ROIs were divided into full-length (contained 3′ and 5′ adapters and poly-A tails before 3′adapters) and non-full-length (did not contain 3′ and 5′ adapters and did not contain poly-A tails before 3′adapters) reads, as well as chimeric (contained two or more transcripts) and non-chimeric (contained only one transcript) reads. The full-length non-chimeric reads were used for clustering, whereas redundant reads were removed. The non-full-length non-chimeric reads were polished with the non-full-length reads to obtain high-quality, full-length isoforms. The full-length isoforms were aligned to the ZS11.v0 reference genome using GMAP v2017.06.20 [76] with min-trimmed coverage = 0.9 and min-identity = 0.85. The mapped full-length isoforms were clustered, and the redundant transcripts were filtered using collapse_isoforms_by_sam.py (https://github.com/Magdoll/cDNA_Cupcake/, accessed on 10 November 2021) with identity < 0.9 and coverage < 0.85. The identification of novel transcripts, fusion genes, and lncRNAs was performed as previously described by Chao et al. [77]. Alternative splicing junctions count was combined with data from the Illumina and PacBio platforms, and they were analysed using SQANTI (https://github.com/ConesaLab/SQANTI/, accessed on 10 November 2021). AStalavista (http://astalavista.sammeth.net/, accessed on 10 November 2021) was used for the AS prediction of full-length isoforms (Figure S14).

### 4.7. Validation by RT-PCR, Quantitative RT-PCR, and Cyclisation RT-PCR 

An EasyScript All-in-One First-Strand cDNA Synthesis SuperMix kit (Transgen, Beijing, China) was used for reverse transcription. LncRNAs, novel transcripts, and fusion genes were identified from cDNA amplification; qRT-PCR was performed using the CFX96 Touch Real-Time PCR (Bio-Rad, Hercules, CA, USA) with the SYBR Green Realtime PCR Master Mix (Biorad, CA, USA). The relative gene expression was determined using the ∆∆Ct method. Each sample was repeated in triplicate. Total RNA was self-linked to a circle using a T4 RNA Ligase (Takara, Tokyo, Japan); then, the circular RNA was extracted with chloroform, precipitated in ethanol, and dried. Reverse transcription was performed with the Reverse Transcriptase M-MLV (RNase H-; Takara, Tokyo, Japan) with the CRT-PCRF and CRT-PCRR2 primers (Table S9). PCR products were detected with agarose gel electrophoresis, whereas TA cloning and sequencing were performed after product recovery (Data S1). A one sample *t*-test was performed on –∆∆Ct for a significant difference comparison.

### 4.8. ROS Analysis of Plant Leaves 

Leaves of 596-L and 1318 and those of 596-L treated at 25 °C for 8 h (596-H) were placed in a 4-nitro blue tetrazolium chloride (NBT) solution (0.1% pH 7.8) for superoxide anion (O_2_^−^) detection. The tissues were vacuumed, decolorised with 95% ethanol, and photographed with a Nikon DS-RI1 camera.

### 4.9. Respiratory Chain Decoupling Treatments 

Mitochondrial uncoupling agent powder carbonyl cyanide 4-(trifluoromethoxy)phenylhydrazone (FCCP, Sigma, Missouri, USA) was dissolved in solubilisers (10% DMSO, 40% PEG4000, 5% Tween-20 and 45% saline) to 10 mM as storage solutions. For respiratory chain decoupling treatments, a preliminary experiment was carried out to determine the optimal concentration of FCCP, that is, 1.5 μL of the FCCP solution (diluted with saline) of different concentrations were dropped on plant leaves, dried, and analyzed for tissue ROS accumulation (NBT was used as the stain). Then, we dropped the FCCP solution in the proper concentration on young buds and the same volume of a blank solvent as a control and took pictures of the buds 4 days later.

## Figures and Tables

**Table 1:** List of candidate genes related to mitochondria.

Gene ID	Description
*BnaA07G0014700ZS*	Mitochondrial outer membrane import complex protein METAXIN
*BnaA07G0016300ZS*	Pentatricopeptide repeat-containing protein
*BnaA07G0019600ZS*	60S ribosomal protein L6
*BnaA07G0027800ZS*	Pentatricopeptide repeat-containing protein
*BnaA07G0028600ZS*	Rhodanese-like domain-containing protein 19, mitochondrial
*BnaA07G0029700ZS*	Mitochondrial phosphate carrier protein 1
*BnaA07G0031500ZS*	Pentatricopeptide repeat-containing protein
*BnaA07G0031700ZS*	Isocitrate dehydrogenase (NAD) regulatory subunit 2
*BnaA07G0033700ZS*	Arabidopsis phospholipase-like protein (PEARLI 4)
*BnaA07G0034100ZS*	Pentatricopeptide repeat-containing protein
*BnaA07G0055400ZS*	Pentatricopeptide repeat-containing protein
*BnaA07G0056400ZS*	Aconitate hydratase 2

## Data Availability

The raw sequence data reported in this paper have been deposited in the Genome Sequence Archive (https://ngdc.cncb.ac.cn/gsa/, accessed on 10 November 2021) with accession numbers CRA004433 and CRA004515.

## Supplementary Materials

The following are available online at https://www.mdpi.com/article/10.3390/ijms222212450/s1.
