# Supplementary material for: Molecular Analysis Uncovers the Mechanism of Fertility Restoration in Temperature-Sensitive Polima Cytoplasmic Male-Sterile Brassica napus
Source: Int J Mol Sci. 2021 Nov 18;22(22):12450. doi: 10.3390/ijms222212450 (PMC8617660; doi:10.3390/ijms222212450)
Supplement: Supplementary file 1 [file ijms-22-12450-s001.zip › ijms-1399011-sup-figure.pdf]

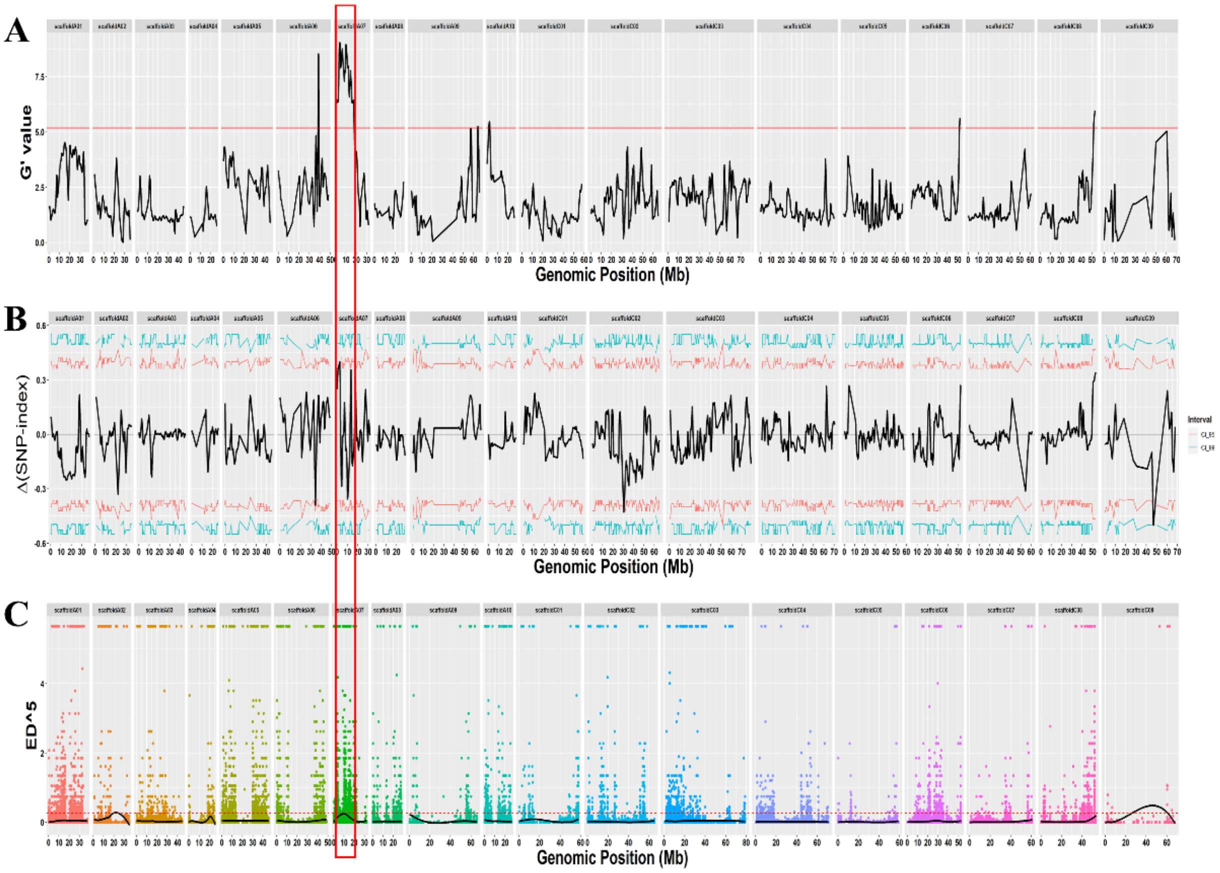

Figure S1. BSA-seq analysis results. The red rectangle represents the predicted candidate interval. (**A**) G′ value method comparison the differences in allele frequencies between the two extreme pools, the red line represents the threshold. (**B**) Delta SNP-index method comparison the differences in allele frequencies between the two extreme pools. (**C**) Euclidean distance method comparison the differences in allele frequencies between the two extreme pools.

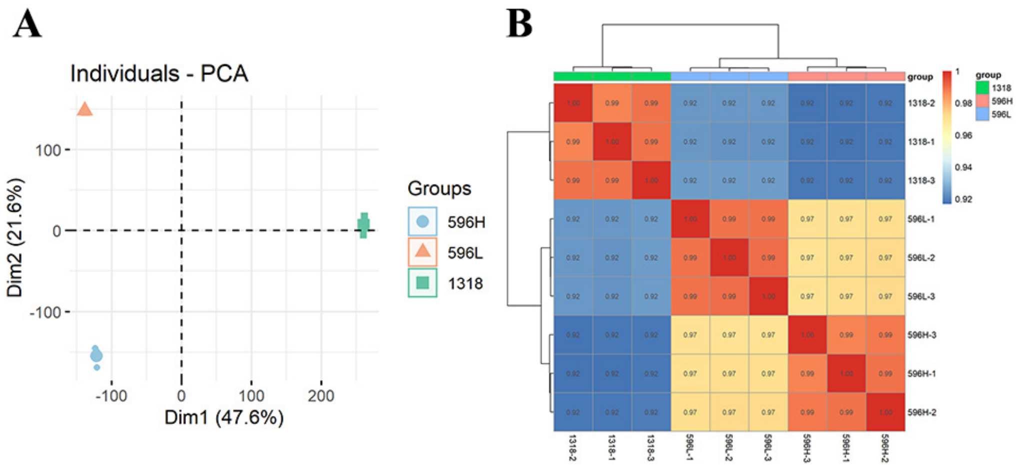

Figure S2. RNA-seq data correlation analysis. (**A**) Principal component analysis between biological replicates. (**B**) Heatmap of expression correlation between biological replicates.

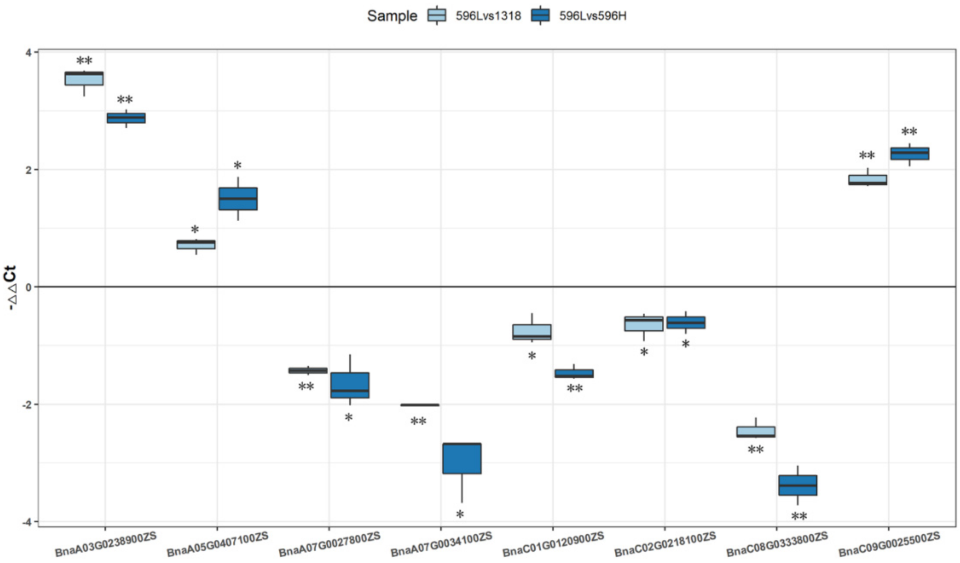

Figure S3. The qRT-PCR validation of gene expression. One sample t-test was used for significant difference comparison, * $p \leq 0.05$, ** $p \leq 0.01$.

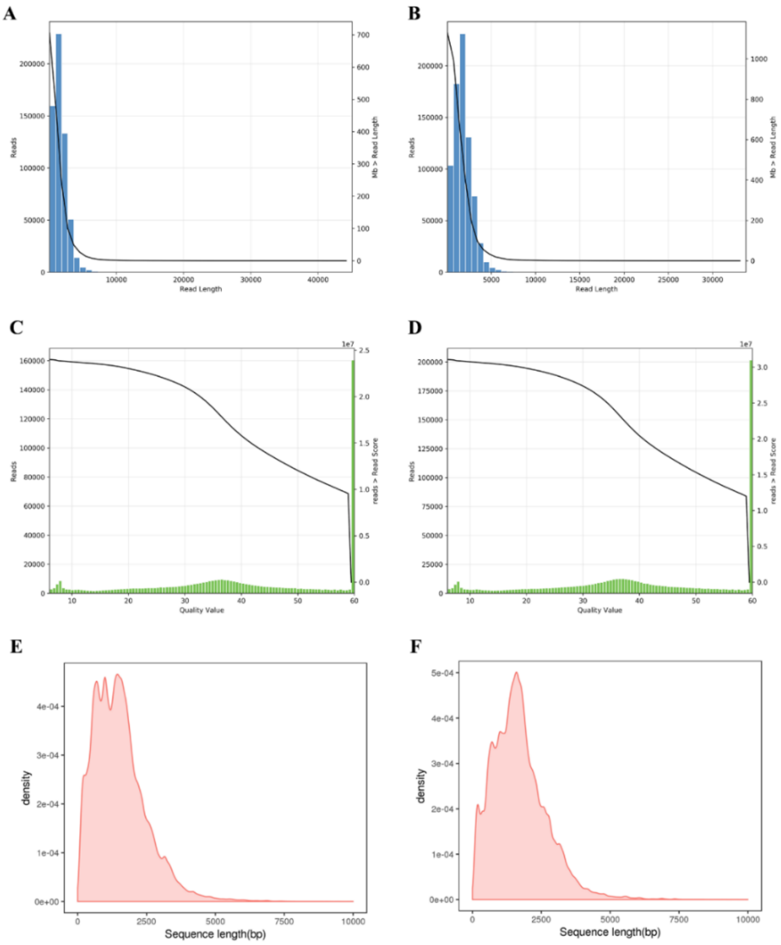

Figure S4. PacBio Iso-Seq read length and quality. (**A**) Distribution of read length in the 1-10 kb 596L library. (**B**) Distribution of read length in the 1-10 kb 596H library. (**C**) Distribution of read quality in the 1-10 kb 596L library. (**D**) Distribution of read quality in the 1-10 kb 596H library. (**E**) Distribution of read length of full-length non-chimeric reads in the 1-10 kb 596L library. (**F**) Distribution of read length of full-length non-chimeric reads in the 1-10 kb 596H library.

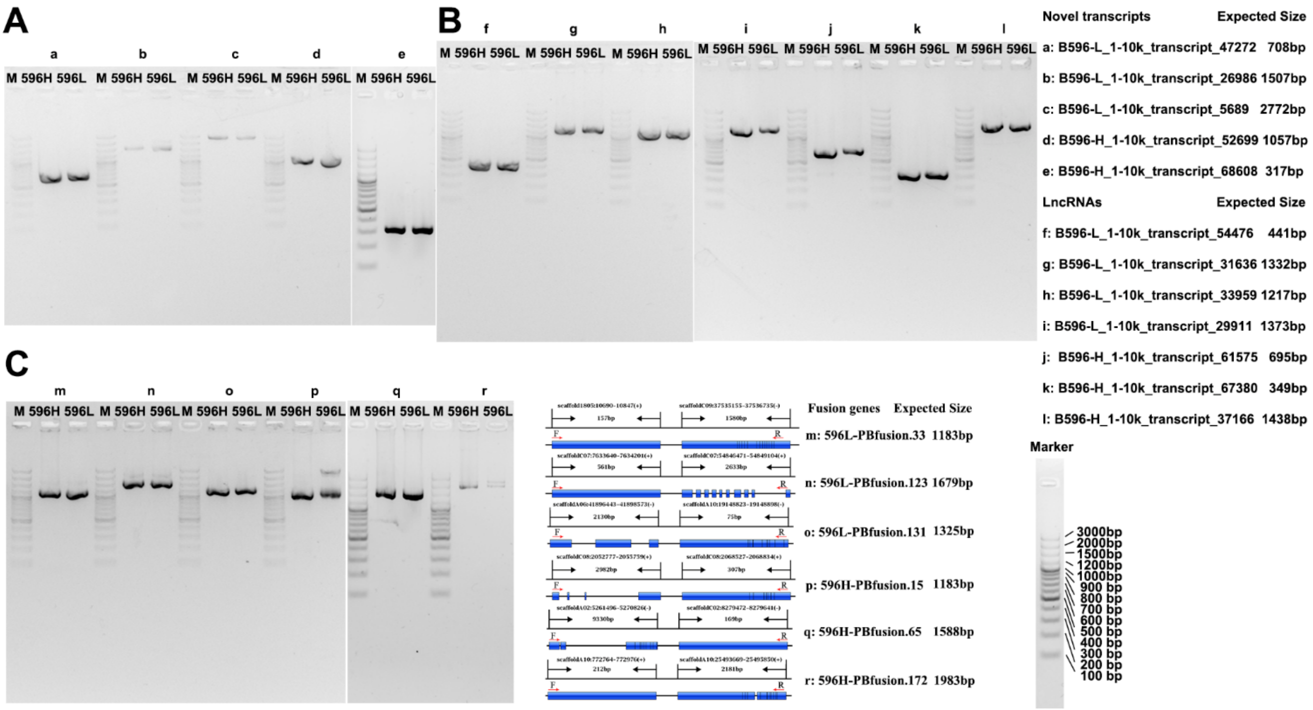

Figure S5. The RT-PCR validation of PacBio Iso-seq isoform. (**A**) The RT-PCR validation of novel transcripts; (**B**) The RT-PCR validation of lncRNAs; (**C**) The RT-PCR validation of fusion genes.

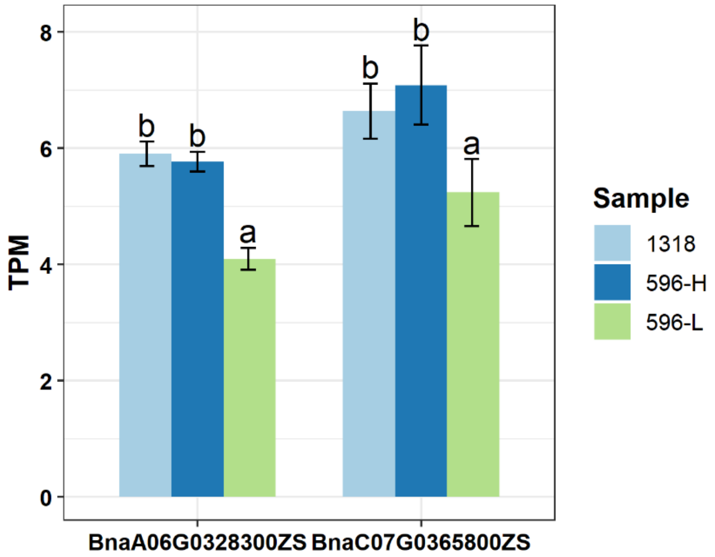

Figure S6. The TPM for *Ire1* gene. Letters indicate significant differences according to one-way ANOVA and Duncan test for post hoc analysis with $p < 0.05$.

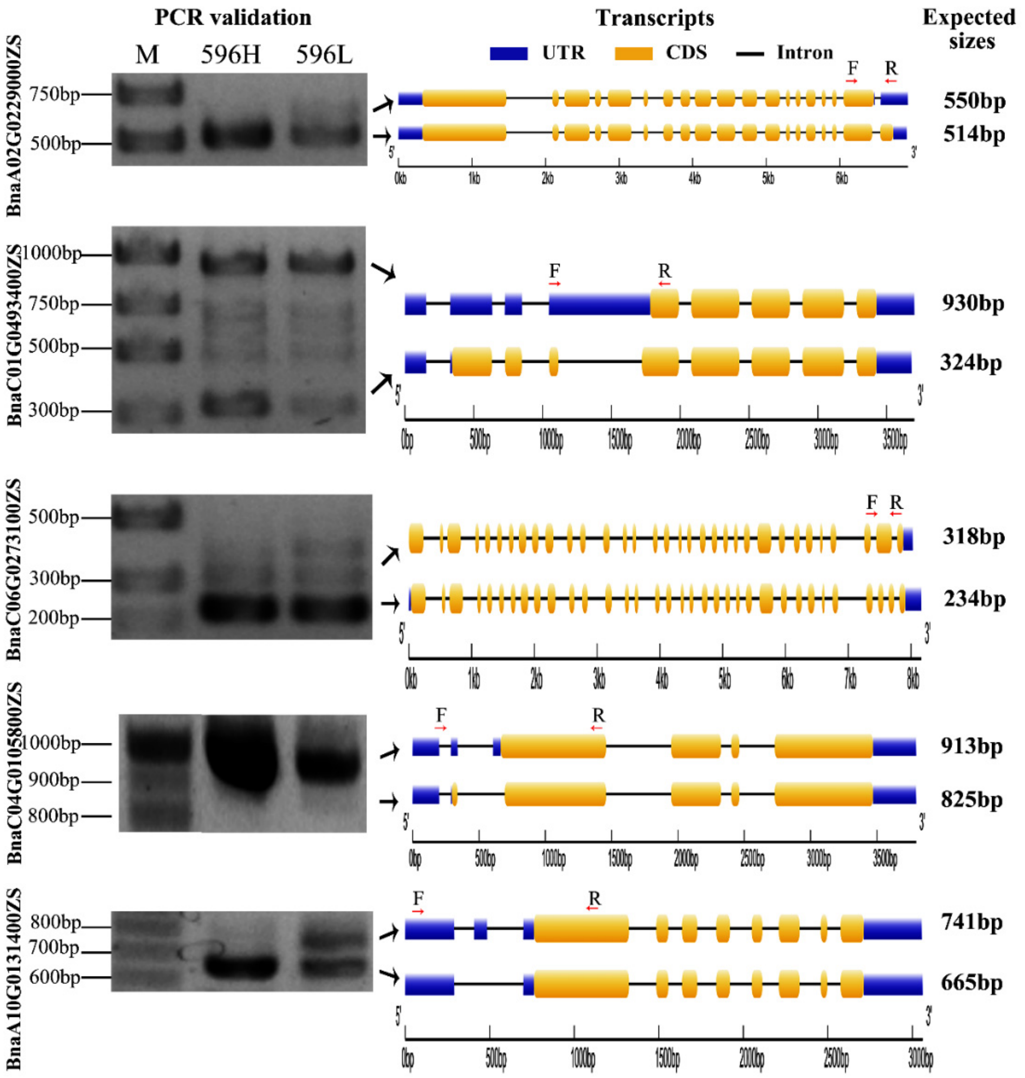

Figure S7. The RT-PCR validation of AS transcripts.

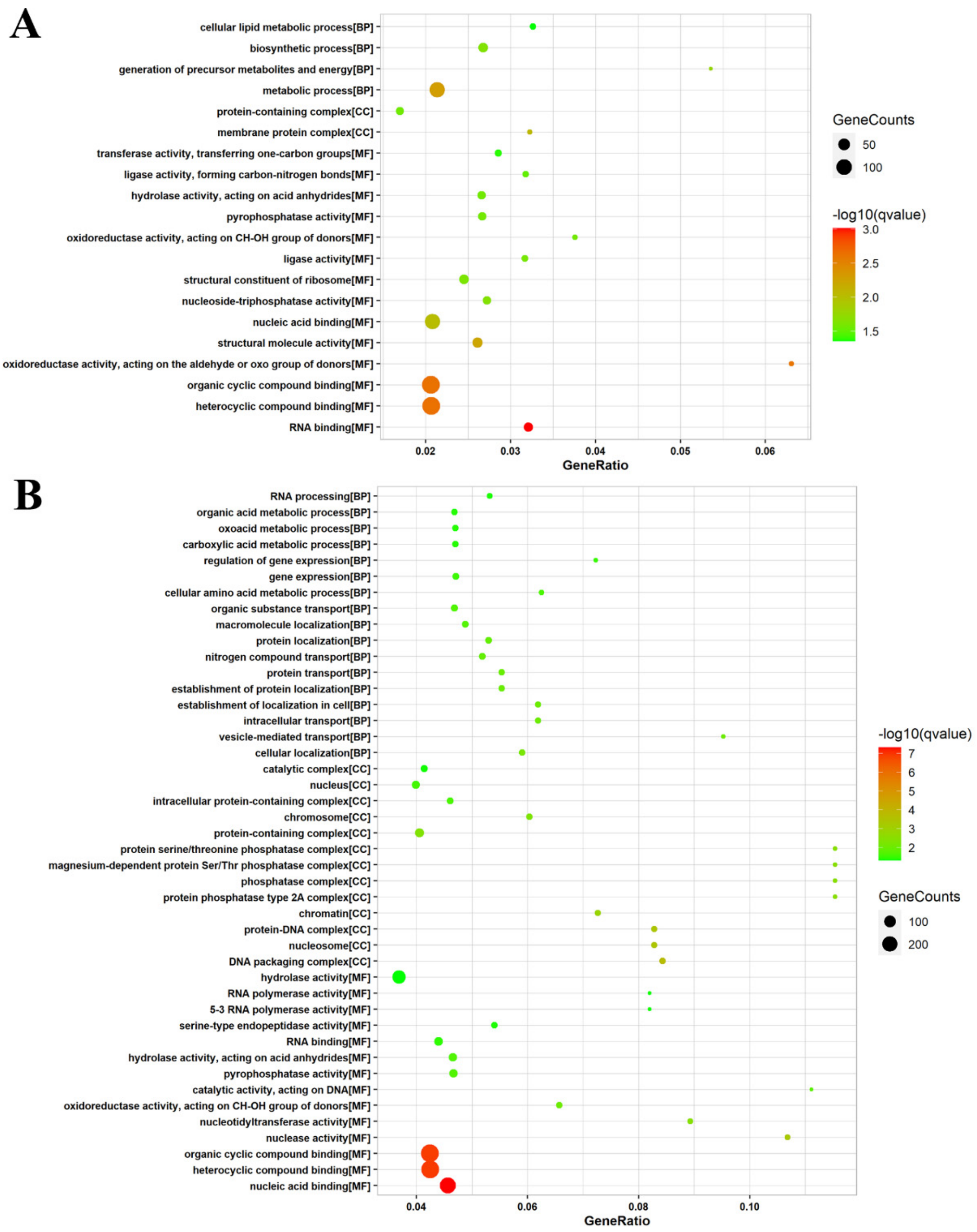

Figure S8. The GO enrichment of specific alternative splicing genes. (**A**) The bubble chart of 596-L; (**B**) The bubble chart of 596-H.

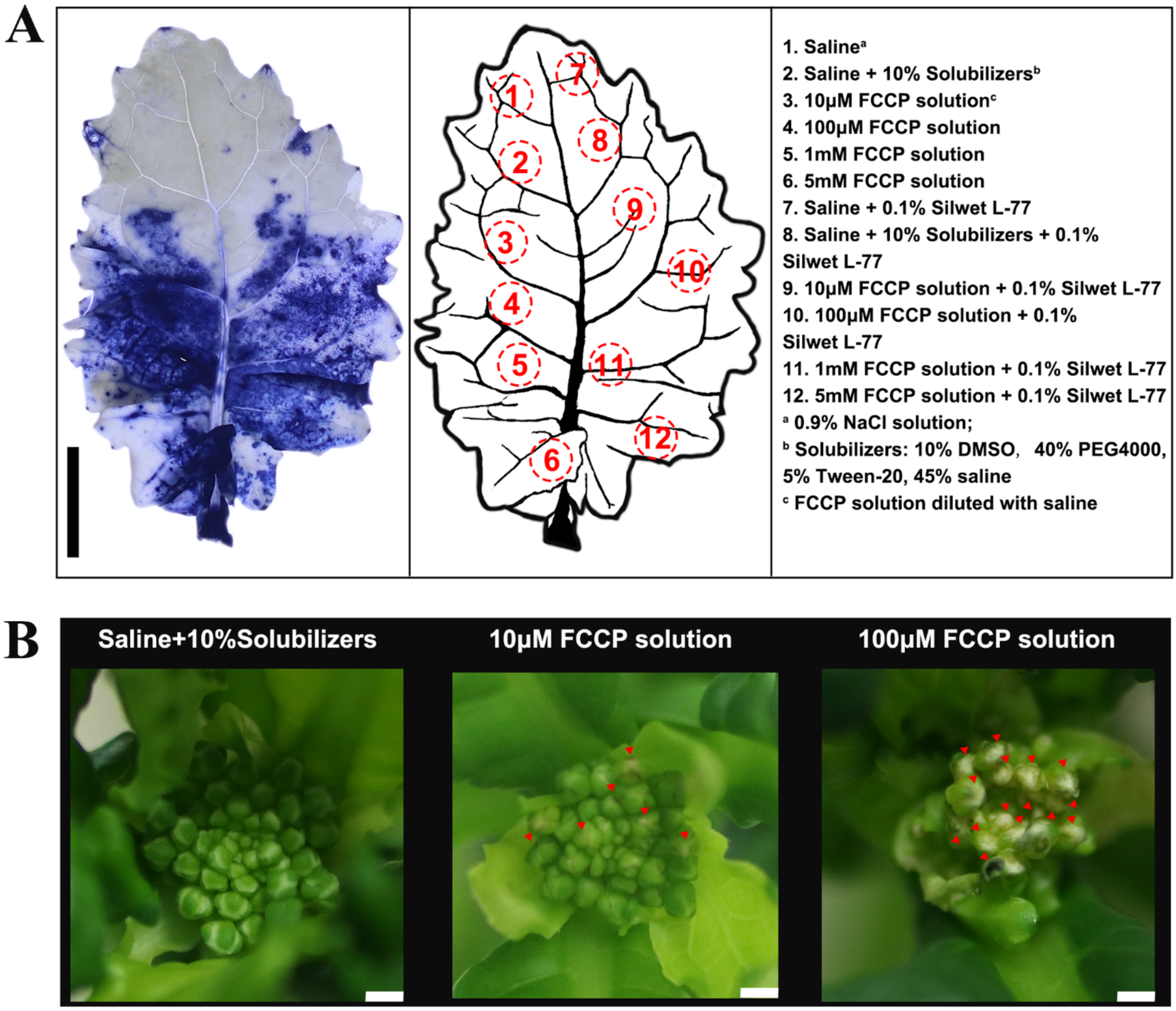

Figure S9. FCCP affects the development of buds. (**A**) ROS accumulation in leaves after FCCP treatment. Bar = 1 cm. (**B**) 596-L young buds' response to different concentration of FCCP solution. Bar = 1 mm.

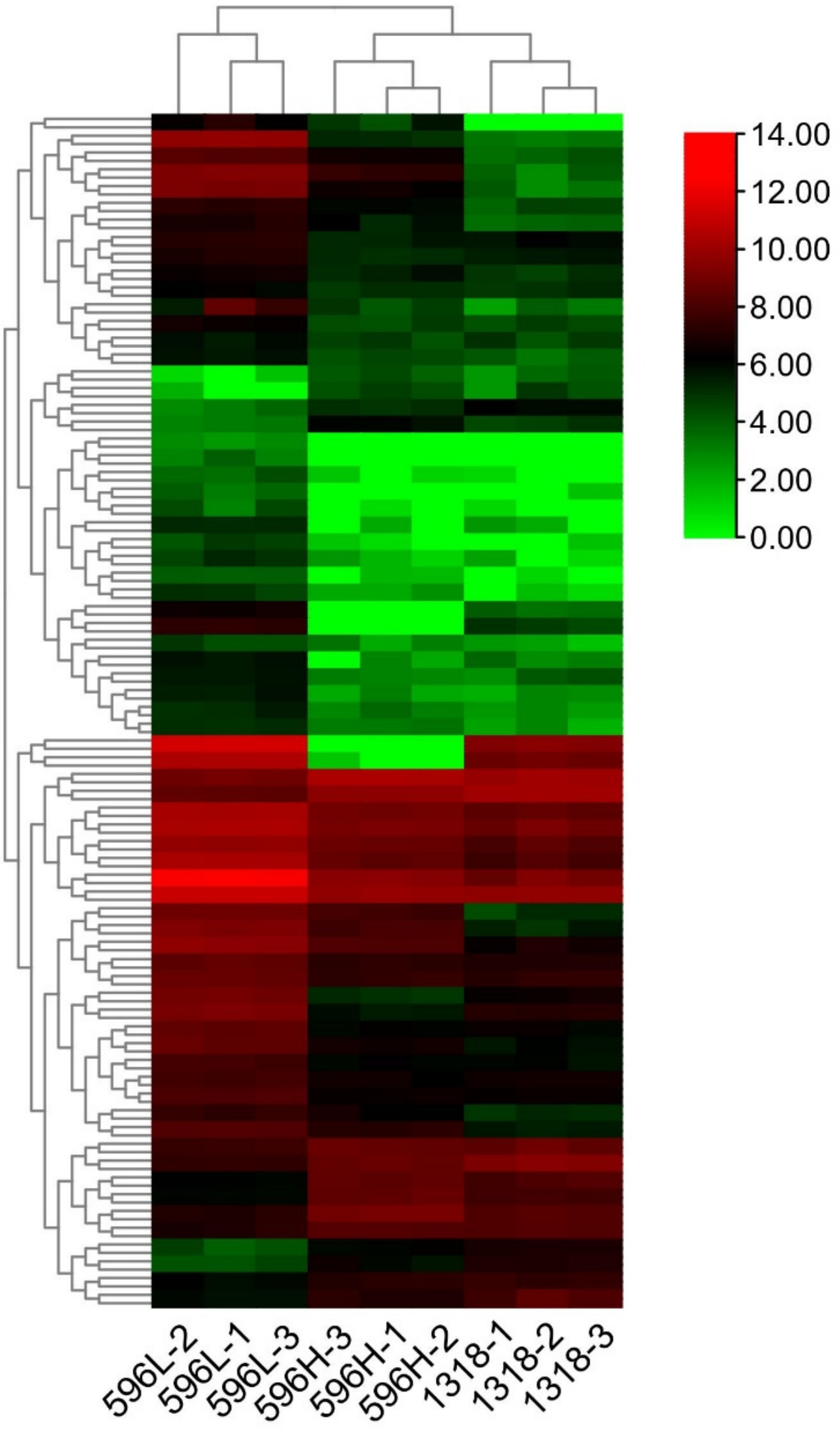

Figure S10. Heatmap showing the DGEs belong to oxidoreductases group.

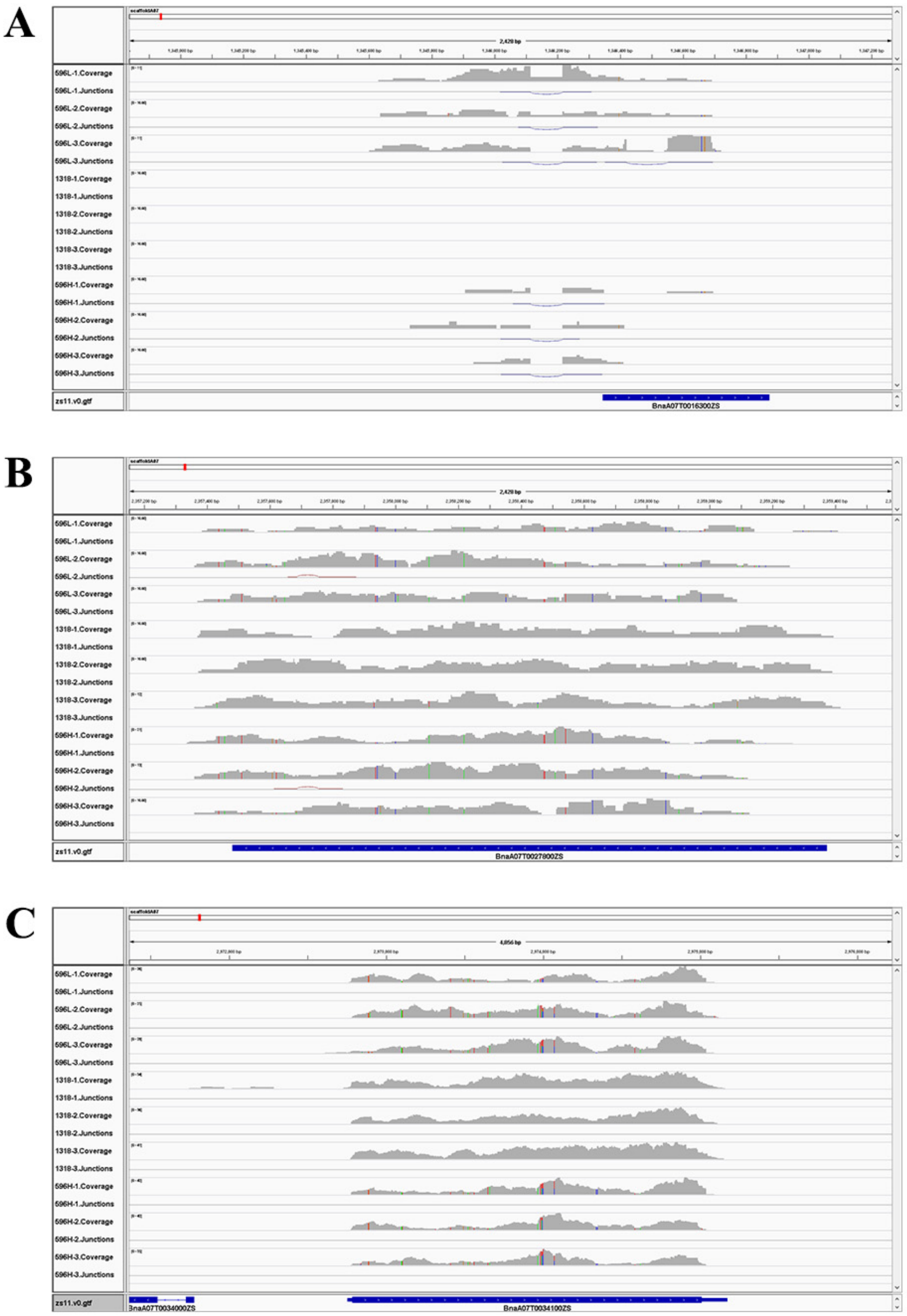

Figure S11. IGV view of candidate gene transcripts. (**A**) BnaA07G0016300ZS; (**B**) BnaA07G0027800ZS; (**C**) BnaA07G0034100ZS.

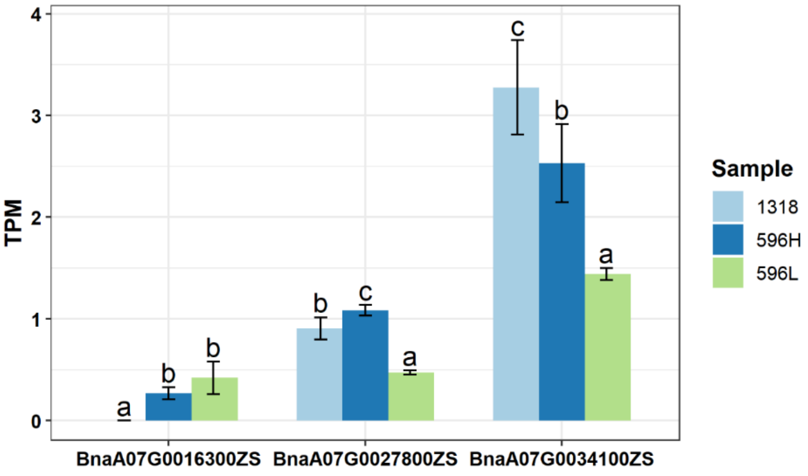

Figure S12. The TPM for candidate gene. Letters indicate significant differences according to one-way ANOVA and Duncan test for post hoc analysis with $p < 0.05$.

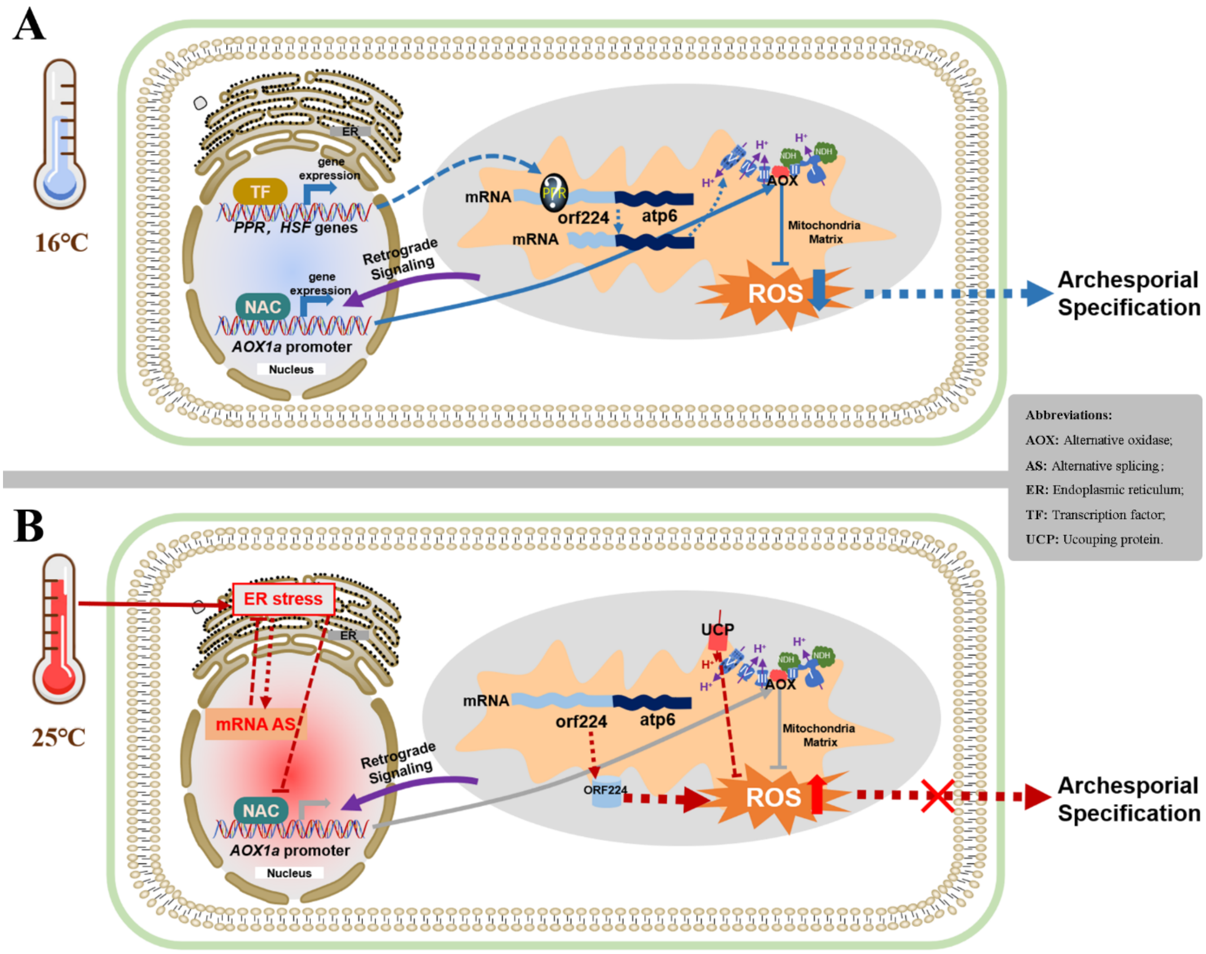

Figure S13. The putative fertility transition model in *pol* TCMS. (**A**) At relatively low temperatures, the co-transcripts of *orf224-atp6* are cleaved by RNA editing factors, allowing ATP synthase to function normally. As a result, the increased energy consumption in the process of anther development elevates ROS levels and activates mitochondrial retrograde signals, *Nac* and some transcription factors that were transferred from the ER into the nucleus upregulated *Aox1a*, activating mitochondrial cyanide-resistant respiration. Furthermore, *Hsfs* is activated to balance redox levels; consequently, hypoxic conditions activate the differentiation of archesporial cells. (**B**) At relatively high temperatures, large number of unfolded proteins or misfolded proteins are accumulated in the cells, leading to ER stress, and RNA editing factors are not synthesised normally, leading to a full-length translation of *orf224* and consequent mitochondrial dysfunction. Besides, transcription factors that activate *Aox1a* cannot fold properly, decreasing the energy consumption in the cyanide-resistant

respiratory pathway. The cells activate the UPR pathway, accelerating the degradation of unfolded proteins. Although the uncoupling of mitochondrial unfolded proteins and ATP synthase helps balance the redox state, ROS accumulation is high in the cells, resulting in the failure of archesporial cell differentiation.

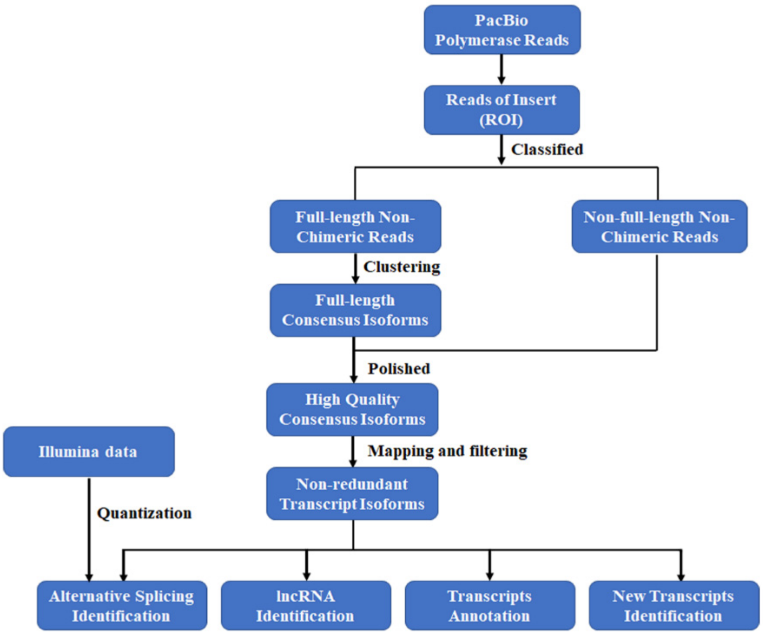

Figure S14. Computational pipeline for joint analysis of PacBio Iso-Seq and Illumina RNA-seq data.
